# Effectiveness of Virtual Medical Teaching During the COVID-19 Crisis: Systematic Review

**DOI:** 10.2196/20963

**Published:** 2020-11-18

**Authors:** Robyn-Jenia Wilcha

**Affiliations:** 1 Faculty of Biology, Medicine and Health University of Manchester Manchester United Kingdom

**Keywords:** virtual teaching, medical student, medical education, COVID-19, review, virus, pandemic, quarantine

## Abstract

**Background:**

In December 2019, COVID-19 emerged and rapidly spread worldwide. Transmission of SARS-CoV-2, the virus that causes COVID-19, is high; as a result, countries worldwide have imposed rigorous public health measures, such as quarantine. This has involved the suspension of medical school classes globally. Medical school attachments are vital to aid the progression of students’ confidence and competencies as future physicians. Since the outbreak of COVID-19, medical schools have sought ways to replace medical placements with virtual clinical teaching.

**Objective:**

The objective of this study was to review the advantages and disadvantages of virtual medical teaching for medical students during the COVID-19 pandemic based on the current emerging literature.

**Methods:**

A brief qualitative review based on the application and effectiveness of virtual teaching during the COVID-19 pandemic was conducted by referencing keywords, including medical student virtual teaching COVID-19, virtual undergraduate medical education, and virtual medical education COVID-19, in the electronic databases of PubMed and Google Scholar. A total of 201 articles were found, of which 34 were included in the study. Manual searches of the reference lists of the included articles yielded 5 additional articles. The findings were tabulated and assessed under the following headings: summary of virtual teaching offered, strengths of virtual teaching, and weaknesses of virtual teaching.

**Results:**

The strengths of virtual teaching included the variety of web-based resources available. New interactive forms of virtual teaching are being developed to enable students to interact with patients from their homes. Open-access teaching with medical experts has enabled students to remain abreast of the latest medical advancements and to reclaim knowledge lost by the suspension of university classes and clinical attachments. Peer mentoring has been proven to be a valuable tool for medical students with aims of increasing knowledge and providing psychological support. Weaknesses of virtual teaching included technical challenges, confidentiality issues, reduced student engagement, and loss of assessments. The mental well-being of students was found to be negatively affected during the pandemic. Inequalities of virtual teaching services worldwide were also noted to cause differences in medical education.

**Conclusions:**

In the unprecedented times of the COVID-19 pandemic, medical schools have a duty to provide ongoing education to medical students. The continuation of teaching is crucial to enable the graduation of future physicians into society. The evidence suggests that virtual teaching is effective, and institutions are working to further develop these resources to improve student engagement and interactivity. Moving forward, medical faculties must adopt a more holistic approach to student education and consider the mental impact of COVID-19 on students as well as improve the security and technology of virtual platforms.

## Introduction

COVID-19 was declared to be a global health emergency by the World Health Organization on January 30, 2020 [1]. The first reported cases of COVID-19 originated from Wuhan City, Hubei Province, in China during the month of December 2019 [1]. Since then, despite stringent global containment measures, including quarantine, testing, and social distancing, the worldwide incidence of COVID-19 has increased rapidly, with a global death toll of 360,679 as of May 29, 2020 [2]. COVID-19 is caused by the novel betacoronavirus SARS-CoV-2; the most common clinical features of the disease include fever, dry cough, chest tightness, and dyspnea [3]. At present, patients with COVID-19 are only treated with supportive care due to the limited use of antiviral drugs [3].

Undoubtedly, one of the countries most affected by COVID-19 is the United Kingdom [2]. As of May 27, 2020, the United Kingdom had reported 268,619 confirmed cases and 37,542 deaths [2]. The public health measures enforced by the UK government center around household isolation [4]. Through government websites and daily televised COVID-19 updates from officials, messages of isolation were reinforced, including staying at home as much as possible, working from home if able, limiting contact with people outside one’s household, and social distancing by remaining two meters apart from others [4].

The impact of COVID-19 on medical education has been substantial. Medical school attachments often require considerable clinical exposure; however, due to the risk of contracting COVID-19, many medical schools in the United Kingdom have discontinued placements [5]. Consequently, students have received decreased exposure to certain medical and surgical specialties, which may in turn reduce the students’ examination performance, confidence, and abilities as future physicians [5]. In these exceptional circumstances, the COVID-19 pandemic has posed an unparalleled challenge to medical schools, which are aiming to deliver quality education to students virtually [6].

The objectives of this study are to review the advantages and disadvantages of virtual medical teaching during the COVID-19 pandemic using the emerging current literature.

## Methods

A systematic review of peer-reviewed literature on the subject of virtual medical education during the COVID-19 pandemic was conducted from May 2020 to June 2020, consistent with PRISMA (Preferred Reporting Items for Systematic Reviews and Meta-Analyses) guidelines [7]. Electronic databases, including PubMed and Google Scholar, were searched using the following key terms: *medical student virtual teaching COVID-19*, *virtual undergraduate medical education COVID-19*, and *virtual medical education COVID-19*. Qualitative results from the review were obtained by comparing and summarizing existing evidence and theories from recent literature.

The quantitative and qualitative studies were chosen based on specific inclusion criteria. The first and foremost criterion was that the study must be published in a peer-reviewed scientific journal. Second, the study was required to present original data assessing virtual medical teaching for medical students, with objectives related to analyzing the effectiveness or perception of this mode of learning. Finally, the included articles reported on studies conducted worldwide between February and June 2020, a period of time central to the COVID-19 pandemic. Due to the shortage of available literature, this review considered any eligible study design, including case reports, case studies, cohort studies, randomized control trials, letters to the editor, commentaries, editorials, and perspectives. The first exclusion criterion was that the article was unrelated to undergraduate medical education. Excluded articles included those focusing on postgraduate medicine and on the teaching of other undergraduate health care professional students, such as dental, veterinary, or nursing students. Moreover, articles that assessed virtual teaching before the COVID-19 pandemic or articles relating to former pandemics were excluded.

The search algorithm yielded 92 articles from the PubMed database and 109 articles from the Google Scholar database. After successful removal of duplicate articles, 185 articles were processed to analyze their titles and abstracts, and a total of 68 articles were found to be eligible for full-text screening. Following the full-text screening, a total of 34 articles were included for data extraction. An additional 5 articles were added after manually searching the reference lists of the included articles. Prominent findings from the review are presented in a table under the following headlines: summary of virtual teaching, strengths of virtual teaching, and weaknesses of virtual teaching.

## Results

**Figure 1:**
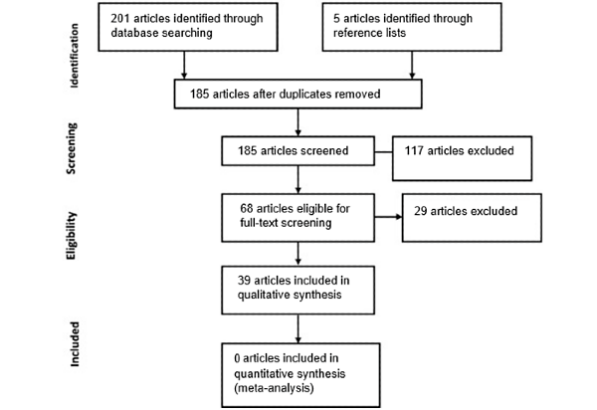
PRISMA (Preferred Reporting Items for Systematic Reviews and Meta-Analyses) flow diagram demonstrating the process of study selection.

In the initial search, 201 articles were found in electronic databases. Following the removal of duplicates, 185 articles were scanned on the premise of title and abstract, and a total of 68 articles were determined to be eligible for full-text screening, of which 34 articles satisfied the inclusion criteria. Manual reviews of reference lists enabled the addition of 5 articles to the review. Figure 1 presents the PRISMA flow diagram, which demonstrates the process of study selection.

The findings from the 39 papers reviewed are tabulated in Table 1. The table documents key findings from original articles relating to the type of virtual teaching offered and the strengths and weaknesses of virtual education during the COVID-19 pandemic. Qualitative analysis of the included articles was conducted.

**Table 1:** Comparison and evaluation of the advantages and disadvantages of virtual education during the COVID-19 pandemic.

Summary of virtual teaching offered	Advantages of virtual teaching	Disadvantages of virtual teaching	Reference
An observational study that reported the use of virtual ward rounds to educate medical students (n=14) regarding COVID-19 cases	It enables direct patient interaction with no risk of infection. It provides insight into a novel disease and active pandemic for medical students. Of the participants, 92.9% strongly agreed that the experience had increased their knowledge and that they were stimulated to learn. Moreover,13 students strongly agreed that they would recommend virtual ward rounds and would continue with this form of teaching. One student remarked that it reconfirmed their motives for studying medicine.	No weaknesses noted	[6]
A letter to the editor that reported the use of web-based education networks for medical students, such as lectures, case discussions, journal clubs, and virtual grand rounds	Immediate access to specialized teaching by medical experts irrespective of geographical location or cost Ease of accessibility Ability to stay up-to-date with the latest medical developments Use of social media as an adjunct to virtual teaching Virtual conferences to accelerate knowledge and interest	No weaknesses noted	[8]
An observational study that reported the use of a medical student response team consisting of 500 students during the COVID-19 pandemic; there were 4 virtual teams that centered around education and activism for both health care professionals and the community	Encouraged development of internal motivation of students while increasing medical knowledge and making a difference Improved team working skills to strive toward a collective goal Students reported feeling empowered and enthusiastic and stated that they had a sense of purpose during the uncertain period of the pandemic	No weaknesses noted	[9]
A reflective study that documented the concerns of medical students regarding their education during the COVID-19 pandemic; the study included 852 students, and 127 responses were analyzed	Virtual mentorship programs and virtual surgical skills workshops were suggested by 67% of medical students, closely followed by webinars (62%) and virtual research symposia (46%).	Loss of networking opportunities Lack of clinical experience Lack of assessments	[10]
A study evaluating the use of virtual medical education platforms	Students could access educational material at their convenience in preferred environments. The study evidenced that virtual reality simulation was as effective as direct patient contact.	Isolation from medical school; reduced interaction and discussion with peers Technical difficulties, including problems with internet access Increased dependence on technology Virtual teaching is costly and time-consuming for faculties, especially if the infrastructure is inadequate Loss of boundaries between work and home Lack of professional development due to absence of influential clinical role models	[11]
A qualitative review documenting the challenges and innovations of virtual medical education platforms	Ease of access with unlimited flexibility Increased learning among medical professionals due to open-access medical resources and virtual conferences Increased interdisciplinary learning to help accelerate evidence-based clinical management Use of virtual interactive technology to promote active, engaging learning Use of social media to promote virtual learning to a wider audience and to offer networking opportunities Increased research and development of simulation programs to allow the continuity of technical skills at home	Loss of clinical opportunities: lack of bedside teaching, lack of direct patient care, halted improvement of examination skills, loss of feedback from tutors	[12]
An observational study that reviewed the use of academic coaching to supplement virtual medical education during the COVID-19 pandemic	Strong support network; collaborative approach between tutor and student Individualized goal-directed study plans with monitoring of study habits and follow-up meetings Increased accountability from students, driving internal motivation Holistic approach that supported students academically as well as mentally, emotionally, and physically	Without academic input, students may have ineffective learning strategies, poor motivation, and suboptimal communication skills, which are maximized by home learning.	[13]
A study highlighting the disruption of anatomy education, from dissecting laboratories to web-based virtual platforms, during the COVID-19 pandemic	No strengths noted	Difficulties understanding anatomy without dissections, practical teaching, or physical aids such as bones, specimens, and models Lack of human visual impact Future scarcity of cadavers due to risk of COVID-19 infection	[14]
A letter to the editor that reflected on the difficulties of virtual medical education	No strengths noted	Difficulties maintaining focus and concentration Costly for facilities providing digital education Inadequate infrastructure to produce a new, functioning virtual medical platform in a short amount of time Inequalities of virtual education created by differences of quality and internet speed Overload of virtual medical platforms	[15]
An analysis of the adaptations made to anatomical education in response to the COVID-19 pandemic. Anatomy education within the United Kingdom has moved away from the use of cadavers to virtual lectures and virtual cadaveric resources.	Opportunity to develop web-based resources Increased academic collaboration between institutions Open access to web-based medical resources to aid anatomical learning	Lack of formal assessments; 50% of universities canceled examinations Time-consuming for facilities to provide virtual platforms with good quality and effectiveness Reduced student engagement with virtual learning; attendance is not monitored Increased risk of isolation, anxiety and boredom Decrease in academic results, quality of life, and motivation as well as increased stress due to lack of social engagement and interactivity	[16]
A study that reported the use of virtual callbacks for patients recently evaluated in the emergency department during the COVID-19 pandemic	Student feedback was positive as a result of patient interaction and improvement of both clinical reasoning and communication skills. The clinical burden on the medical team decreased. Patients were reassured by receiving follow-up after discharge from the emergency department.	No weaknesses noted	[17]
A study conducted in Nepal evaluating the use of virtual medical education platforms	The broader audience allows increased learning compared to the traditional classroom. Web-based classes at set times hold students accountable for their learning.	Requires all students to have a reliable internet connection and use of digital devices Lack of knowledge on how to operate virtual platforms Difficult to retain concentration while looking at a screen for long hours Difficulties finding a quiet and private learning environment	[18]
A study that evaluated the use of virtual morning reports to deliver effective virtual teaching during the COVID-19 pandemic	Enables development of clinical reasoning skills Interaction between clinician educators, active medical students, and passive medical students, enabling immediate feedback Supportive learning environment between peers and teachers Ease of accessibility due to asynchronous viewing and multi-institution participation	“Zoom bombing:” hackers can invade Zoom sessions, creating potential security breaches. On occasion, critical and disrespectful comments were made by other Zoom users.	[19]
A study encouraging the sharing of virtual learning materials between institutions to aid virtual undergraduate medical education	Rapid development of engaging and good-quality virtual learning materials More robust content	No weaknesses noted	[20]
A study evaluating the use of web-based virtual platforms for medical students and the future role these platforms may play in medical education after the COVID-19 pandemic	Increased class attendance due to ease of access Increased student engagement due to student anonymity within sessions Increased number of medical webinars to accelerate the exchange of ideas	Technical difficulties, such as configuring hardware and software Time-consuming and costly for faculties	[21]
A letter to the editor that explored how to sustain learning during the COVID-19 pandemic via the use of webinars, case-based discussions, journal clubs, and virtual classrooms	Flipped classroom style of learning, allowing development of problem-solving skills, critical thinking, and self-directed learning Accessibility of learning from experts around the globe. Flexible learning Increased research conducted during the pandemic Increased personal development, such as resilience, during the pandemic	Loss of clinical and surgical skills Technical difficulties of virtual learning, such as reduced internet speed and quality Not all students may have access to digital technology Reduced student engagement; lack of focus, multi-tasking with other activities, and poor audio and video quality Lack of physical, mental, and social support from peers and institutions; anxiety may hinder learning Lack of formal assessments due to lack of security and validity	[22]
A pilot study that reported the use of virtual clerkship for medical students (n=6) for 14 days.	Advancement in medical knowledge and clinical reasoning skills through social learning and cognitive apprenticeship Interactive sessions between teachers, students, and peers increase student engagement Feedback provided by tutors to aid progression of studies Use of virtual ward rounds to reinforce learning from independent study, podcasts, and conferences Five of the six students provided positive feedback for the virtual clerkship and stated they would continue with this form of teaching	It is time-consuming for clinicians to aid learning in light of extended work service due to the COVID-19 pandemic. Virtual clerkship is not easily scalable to encompass all medical students.	[23]
A study conducted in Iran documenting the shift to virtual medical education	Ease of accessibility due to asynchronous learning Opportunity to enhance virtual medical education for the future	Lack of preparation and inadequate infrastructure for virtual learning Impossibility of training for all age groups within the medical curriculum Inability to virtualize every aspect of a medical course	[24]
A study evaluating the use of virtual medical education for medical students	Continuous learning despite the pandemic Increased flexibility and accessibility to learning Synchronous virtual sessions enable excellent communication between teacher and student Complete digital access to world-class experts at any time	Technical challenges, especially with audio and video Issues with confidentiality and security	[25]
A study demonstrating the value of peer learning during the COVID-19 pandemic	Peer discussion facilitates active discussion, sharing of ideas, critical thinking, and collaboration Enhancement of motivation, teamwork, conflict resolution, and task management Peer learning reduces stress and develops resilience Increased examination performance documented for April 2020 due to improvement in problem solving	No weaknesses noted	[26]
A pilot study comparing face-to-face and virtual teaching of surgical skills for final year medical students (n=30)	40% of students highly recommended virtual teaching (score of 8-9/10) 50% of students slightly recommended virtual teaching (score of 6-7/10) Affordable manner of teaching surgical skills	Recorded sessions removed teacher-student interaction Difficulty learning intricate surgical skills due to limited camera angles Technical difficulties accessing web-based platforms	[27]
A study highlighting the possible methods of virtual education, including modules, reading assignments, and virtual scenarios	Continuous education during the pandemic Use of social media, specifically Twitter, to promote virtual learning	No weaknesses noted	[28]
A letter to the editor documenting the structural changes of medical education within Brazil	No strengths noted	No infrastructure for virtual education; investment in infrastructure is challenging for a developing country. Students may not have access to digital technology. Students may be socially vulnerable, increasing the challenges of educational activities.	[29]
A study conducted in Nepal demonstrating the difficulties faced by virtual medical education	Continuous education during the pandemic	Lack of accessibility to virtual platforms as a result of difficulties establishing internet connection (cost, quality, and speed) and difficulties obtaining digital devices Sense of isolation decreases student participation and may cause student withdrawal; lack of socialization is linked to decreased academic achievement and mental distress Difficulty for tutors to assess student disengagement, frustration, or disinterest No pressures for students to attend classes or access learning materials Physical discomfort from virtual learning, such as exhaustion, visual discomfort from looking at screens, and muscle or joint pains from remaining stationary	[30]
A letter to the editor documenting the impact of COVID-19 on the medical curriculum; the article references the effectiveness of Zoom and web-based lectures	Zoom is a highly effective virtual learning tool with reports of high student engagement. Web-based webinars are used to cover relevant material in a “bite-size” manner. Feedback from students was positive, with a regular number of medical students attending.	No weaknesses noted	[31]
A letter to the editor reflecting on the loss of clinical opportunities faced by current medical students	No strengths noted	Students must spend time on the ward with direct patient contact to prepare for the realities of working life.	[32]
A study evaluating the use of digital clinical placements in response to the COVID-19 pandemic	Weekly set of interactive web-based cases supplemented by patient videos to increase student engagement and exposure to a variety of conditions Development of clinical reasoning	Limited access to patients	[33]
A study evaluating the impact of virtual education on current medical students during the COVID-19 crisis.	Gentle impact on preclinical medical students due to the normal lecture format of teaching Open access to medical resources during the pandemic, aided by social media promotion Use of Zoom as a highly effective tool for virtual learning	Inadequate preparation for preclinical medical year students; no teaching of history taking or physical examinations, which are building blocks for the clinical years Loss of clinical placements may affect ultimate specialty choice Virtual learning can be time-consuming for clinicians, especially in times of uncertainty and increased demand Difficulties in virtually assessing audience understanding and interest Increased stress of balancing home life and work life, with little separation between the two	[34]
A study documenting the changes in medical education within the United Kingdom; the study references virtual teaching at Imperial College London, where patients are interviewed virtually by both physicians and medical students to facilitate teaching	Excellent student attendance and interaction Identification of a variety of pathologies, signs, and symptoms through patient interviews, which develops clinical reasoning skills and diagnostic thought processes No exposure to infection despite patient contact. Reduced burden on the health care system by providing an effective triage service	Students reported a decline in confidence in their skills while conducting virtual learning. Preclinical medical students were adversely affected due to a lack of clinical foundation.	[35]
A study that reported the effects of virtual medical education on students in Italy	Virtual learning is effective to achieve primary aims and continue education in the short-term.	Long-term virtual learning would have negative effects on students, administrative staff, and tutors.	[36]
A study that documented the replacement of clinical general practice attachments with e-learning programs in Australia	The study found that web-based learning is as effective as traditional teaching. Students could submit a web-based learning portfolio to enable accurate assessment as well as to demonstrate competency.	Technical difficulties can hinder learning. Virtual learning may compete with other responsibilities. Sharing of technology may hinder learning. Virtual learning may not provide a medical student with a full skill set.	[37]
A letter to the editor reflecting on student perspective and feedback regarding undergraduate ophthalmology virtual learning during the COVID-19 pandemic	97.2% of students felt that web-based classes were a viable alternative to classroom lectures. 84.7% of students were familiar with web-based virtual learning platforms. Learning was easily accessible. Students could review information to aid learning.	Reduced interaction in comparison to classroom teaching Increased doubts relating to knowledge Internet difficulties relating to poor connection or unavailability of digital technology	[38]
A study evaluating the student perspective of e-learning during the COVID-19 pandemic; a survey was sent to 983 students in April 2020 questioning the effectiveness and satisfaction of web-based classes	Students reported that virtual teaching was as effective as classroom teaching for improving communication, building skills and knowledge, preparing for their professional career, and submitting assignments. Students were satisfied with the availability of electronic resources being offered.	Students found that virtual teaching was less effective than classroom teaching for convenience, interaction, understanding individualized learning needs, and balancing practical and theoretical skills.	[39]
A study that demonstrated the effectiveness of virtual OSCEs^a^ during the COVID-19 pandemic; a teleOSCE^b^ was performed through Zoom with 49 medical students	There was no difference in mean score (mean difference –1.1; 95% CI –2.8 to 0.7; *P*=.2) or failure rate (rate difference 2%; 95% CI 0.7% to 10.7%; *P*=.06) between the groups.	No weaknesses noted	[40]
A commentary discussing the transition of medical education to the internet	Use of flipped classroom style teaching before COVID-19; sense of familiarity for students Latest medical research available on the internet	Lack of formal assessments	[41]
A study that evaluated the use of virtual pastoral support during the COVID-19 pandemic	The service was convenient to use on smartphones and computers. Students readily adapted to the service; levels of student engagement were high. Peer support groups, facilitated by staff, were found to have a positive impact through student feedback; the groups offered relief of stress and respite from studies.	Technical difficulties, such as poor internet connection and lack of access to technology or bandwidth Staff cautious of virtual pastoral support due to general discouragement of social media contact and mobile phone contact with students Virtual learning increased social withdrawal, contributing to anxiety and loneliness	[42]
A study that documented the web-based transition of MCQs^c^ for a medical education faculty	Use of Zoom with a web-based coach to help facilitate designing online MCQs; higher engagement in virtual sessions than in face-to-face workshops Self-reflection on the quality of their own written MCQs Newly designed MCQs in adjunct with the virtual workshops were higher in quality than previous MCQs	No weaknesses noted	[43]
A review noting the impact of COVID-19 on medical education as well as mental well-being	Virtual learning is not new; many faculty members had prior training in the use of web-based platforms.	Demand for computers and information technology equipment came from students and families alike. Assessment of virtual technology is underdeveloped; change of assessment structure, students may cheat. Some forms of assessment, such as laboratory practical assessments, are impossible to conduct on the internet.	[44]
A study that reported on the effectiveness of peer mentoring for medical students (n=371) during the COVID-19 pandemic via the use of WhatsApp	71% of junior medical students felt that mentoring helped them adjust more rapidly to the COVID-19 crisis. Senior medical students reported that the experience enabled significant professional growth.	Students desired face-to-face social interaction despite virtual interaction	[45]

^a^OSCE: objective structured clinical examination.

^b^teleOSCE: teleconferencing objective structured clinical examination.

^b^MCQs: multiple choice questions.

## Discussion

### Summary of Results

This exploratory review questions the effectiveness of virtual teaching for medical students during the COVID-19 crisis by comparing the advantages and disadvantages listed in all available literature reports, as documented in Table 1.

#### Principal Strengths of Virtual Teaching

The COVID-19 pandemic has provided medical education institutions with a unique opportunity to adapt and advance their medical teaching methods. Previously, medical institutions relied upon classroom teaching, such as lectures, for preclinical year medical students, followed by various specialty medical attachments, completed in hospitals, for clinical year medical students [35]. As a result of the COVID-19 pandemic, university studies were suspended, and a rapid transition to virtual learning occurred [35].

The analysis of the available literature reveals several advantages of virtual teaching for medical students. First, the development of interactive virtual clinical teaching has been shown to be one of the most effective forms of virtual teaching, ranked by advancement of knowledge, student engagement, and student feedback. Hofmann et al [6] explored the use of virtual ward rounds to allow medical students to observe and interact with patients with COVID-19 while eliminating the risk of infection. Although their sample size of 14 students was limited, Hofmann et al demonstrated that students were enthusiastic to learn about a novel disease that is directly relevant to the world. Through student feedback, it was found that 92.9% of students recommended this form of teaching and agreed that it stimulated learning [6]. Similarly, a study by Murdock et al [19] evaluated the use of virtual morning reports to deliver effective and engaging teaching to medical students from multiple institutions worldwide. The strengths of this mode of teaching included the active development of student clinical reasoning skills as well as the ability to gain feedback from tutors and peers alike. Some institutions developed virtual clerkships, via the use of Zoom, to further increase medical students’ clinical exposure. Chandra et al [17] reported on the use of virtual callbacks conducted by medical students for patients who had recently evaluated in the emergency department. This program was found to help all parties; student feedback was overwhelmingly positive as a result of the direct patient interaction, and the students further stated that they felt that this mode of teaching increased their clinical reasoning and communication skills. Moreover, there was a reduced clinical load for the medical team, and the patients found it comforting to receive a follow-up appointment upon discharge. Likewise, a pilot study from Geha et al [23] reported on the use of 14-day virtual clerkships for medical students. The results of this paper indicated an advancement in medical knowledge and clinical reasoning skills through social learning and cognitive apprenticeship, increased student engagement due to interactivity, and the ability to learn from real-life patients. Although the study was limited due to a sample size of 6 students, the study showed that 5 of the 6 students felt positive about the placement and wanted to continue with this form of teaching above others [23]. Furthermore, Imperial College London hosted virtual patient interviews for medical students; the study reported excellent student attendance and interaction, with benefits of increasing student clinical reasoning skills and diagnostic thought processes as well as reducing the burden on the health care system by providing an efficient triage system [35]. Finally, Harvard Medical School developed the use of virtual medical student response teams that consisted of 500 students arranged into 4 virtual teams with the aim to either educate or help the community or health care teams [9]. Due to the active role of helping during a worldwide pandemic, students reported feeling empowered and enthusiastic, and they felt a sense of purpose during uncertain times. Moreover, the project facilitated team working skills and indirectly increased student knowledge and awareness of COVID-19 [9]. Despite the highlighted successes of these highly interactive forms of virtual teaching, limited literature is available on these programs, suggesting that they are underdeveloped and not in use by most medical education facilities. Potential factors that may contribute to the underdevelopment of these specific programs are the scalability of the programs as well as the time commitments needed from clinicians when work demand is already at a critical stage [23].

The main format of virtual teaching for medical students is through virtual web-based platforms. Virtual web-based platforms consist of webinars, case discussions, reading assignments, and prerecorded virtual scenarios [28]. An advantage, noted through multiple publications available on this theme, was the ease of accessibility and unlimited flexibility of medical resources [8,11,12,19,22,24,25]. In a time of great uncertainty and doubt, providing students with increased flexibility and access to teaching materials may further encourage self-directed learning and motivation. Furthermore, open access teaching from world-renowned medical specialists, irrespective of location and cost, has now become available during the COVID-19 crisis [8]. Teaching provided by experts and promoted through social media platforms such as Twitter and Instagram Live can act as valuable adjuncts to virtual teaching. This teaching can also accelerate student knowledge and interest in specialties a student has not yet experienced and can enable students to observe the latest medical advancements [8,12,28,34]. Networking between students and physicians, which many students thought would be lost due to the suspension of attachments, has been revived through the use of virtual conferences and social media [8,12].

Peer mentoring during the COVID-19 crisis has proved to be a valuable form of teaching. Peer mentoring involves student-to-student teaching; it helps develop active discussion, exchange of ideas, critical thinking, and collaboration between colleagues [26]. In uncertain times such as the COVID-19 pandemic, peer mentoring can help drive student motivation and task management, increasing the effectiveness of self-directed study [26]. Personal development may also be improved by peer mentoring; qualities such as resilience, conflict resolution, and leadership may all be developed through this mode of teaching [26]. A study by Mohammed Sami Hamad et al [26] demonstrated that peer learning may also lead to increased examination performance due to improvements in problem-solving skills. Peer mentoring can also be used to provide psychological support to colleagues. A study by Rastegar Kazerooni et al [45] discussed the use of a WhatsApp group consisting of 371 medical students to provide advice and reassurance during the pandemic. Using student feedback from the study, it was found that 71% of junior medical students reported a smoother transition with quicker adjustment to the COVID-19 crisis, and senior students benefited from significant professional growth [45].

Student perception of virtual teaching is imperative to understand to deliver effective teaching throughout the pandemic. A study conducted by Sud et al [38] reported that 97.2% of students felt that web-based classes were a good alternative to classroom teaching during the pandemic. This was further reiterated by a study by Kaur et al [39] that surveyed 983 medical students on their satisfaction with virtual teaching during the COVID-19 crisis. The outcomes of the study showed that students felt that virtual teaching was as effective as classroom teaching for improving communication, increasing knowledge and skills, professional growth, and submission of assignments. Moreover, students were happy with the availability of electronic resources offered by virtual learning platforms [39]. Upon review of the current literature, it is evident that medical students have a strong passion and determination to learn during the pandemic. Articles written by Sandhu et al [21] and Marques du Silva [31] demonstrated increased class attendance at webinars and positive student feedback of web-based extracurricular lectures. Guadix et al [10] conducted a survey to understand what medical students with an interest in neurosurgery desired from virtual teaching. Of the 127 students who responded, 67% wanted virtual mentorship programs and virtual surgical skills workshops in addition to their medical school studies [10].

#### Principal Weaknesses of Virtual Teaching

A significant number of published studies indicated that a major disadvantage of virtual teaching is technical difficulties [11,15,18,21,22,25,27,29,30,37,38,42,44]. On further analysis of the technical difficulties experienced by students, several different challenges to virtual teaching arose. The largest problem presented by virtual teaching was that some students had no access to digital technology; thus, virtual learning was an ineffective or impossible form of teaching for those students [22,29,30,38,42,44]. Other technical challenges included difficulties establishing a reliable internet connection [11,18,27,30,38,42], problems with hardware and software for virtual learning platforms [21,42], problems relating to internet speed and quality [22,37], and problems with audio and video playback [25]. Moreover, a study by Machado et al [15] reported that virtual learning platforms may become overloaded due to the sheer number of students accessing the materials; overload of a platform stops the platform from working and hinders student learning [15].

Papers by Murdock et al [19] and Sleiwah et al [25] raised concerns with respect to confidentiality and security issues [19,25]. “Zoom-bombing” is a practice in which hackers invade Zoom sessions; therefore, virtual sessions that document real patient information may be at risk of security breaches [19].

The loss of face-to-face teaching was another significant weakness of virtual teaching. The loss of clinical attachments was referenced by numerous publications [10,12,22,34]. Dedeilia et al [12] suggested that the loss of clinical attachments, subsequently causing a loss of bedside teaching, a lack of direct patient care, and a loss of feedback from clinicians, halted the progression of the competencies of a medical student. This was further reiterated by Kaup et al [22], who reported a decline in the clinical and surgical competencies of medical students during the pandemic [22]. Interestingly, Sahi et al reported a possible cessation of professional growth of medical students due to a lack of influential clinical role models during this time [11]. Furthermore, Hilburg et al [34] described a life-changing effect in which the loss of clinical attachments during medical school may impact the specialty the student chooses to pursue in later life [34].

The transition to virtual teaching via the use of web-based medical education platforms presents its own individual disadvantages. Studies by Machado et al [15] and Atreya et al [18] reported the hardships of maintaining focus and concentration whilst sitting in front of a screen. Similarly, Lee et al [13] found that without academic input, students were more likely to have ineffective learning strategies, poor motivation, and reduced communication. Physical discomfort, such as exhaustion, visual problems, and muscle and joint pain, was also reported with long periods of virtual teaching [30]. Unsurprisingly, papers by Longhurst et al [16] and Kaup et al [22] found reduced student engagement levels associated with virtual teaching. Longhurst et al [16] suggested that student engagement decreased as a result of reduced monitoring of students, whereas Kaup et al [22] argued that reduced student engagement was due to a lack of student focus, interest in other environmental activities around students, and technical difficulties [22]. Surkhali et al [30] highlighted that a further disadvantage of virtual teaching is that tutors have difficulties assessing student disengagement, frustration, and disinterest; this may reduce the quality and effectiveness of virtual teaching. Loss of student-tutor interactivity was another potential causative factor for reduced student engagement, as evidenced by Michael et al [27] and Sud et al [38].

Concerns regarding preclinical year medical student education were also identified in this review. Studies by Hilburg et al [34] and Mian et al [35] highlighted that this student cohort would have significantly weak clinical foundations, which provide the building blocks for students’ clinical years and subsequent life as physicians. Hilburg et al [34] argued that the lack of face-to-face teaching for skills, such as history taking and physical examinations, would negatively impact students’ transition to their clinical years. Furthermore, suspension of studies has drastically disrupted the teaching of anatomy. A paper by Singal et al [14] documented the difficulties faced by students in understanding anatomy without the tools of dissection, practical teaching, specimens, or slides. A future concern of anatomists and medical education facilities alike is a lack of cadavers following the COVID-19 pandemic due to the potential risk of infection of the deceased [14].

The loss of formal assessments is an additional weakness of virtual teaching. A study by Longhurst et al [16] reported that 50% of medical student examinations had been cancelled, and even more had been adjusted to unfamiliar formats. Kaup et al [22] argued that this was due to a lack of security and validity of conducting virtual examinations. Interestingly, a study published by Lara et al [40] documented the novel use of a teleconference objective structured clinical examination (teleOSCE) during the COVID-19 pandemic. The results of this study indicated that for the 49 medical students who participated, there was no difference in mean score or failure rate between face-to-face and virtual OSCEs, suggesting that this form of assessment was an effective and reliable method of testing and could be explored in the future [40].

Weaknesses of virtual teaching identified by medical students included reduced interaction between peers and tutors, reduced understanding of individualized learning needs by tutors, and the difficulties of balancing practical and theoretical skills [39]. Virtual teaching platforms from the perspective of medical education faculties were found to be costly and time-consuming [11,15,16,21].

#### The Use of Virtual Medical Education Worldwide

The literature included in this review includes papers written worldwide. Upon analysis, it is evident that virtual teaching for medical students differs by country and that students may have exceedingly different learning experiences [9,18,24,29,30,35,37]. These different learning experiences delivered by virtual teaching may create inequalities of knowledge, confidence, and skills of medical students on a global basis [15].

In developed countries, such as the United Kingdom, Italy, the United States, and Australia, virtual teaching for medical students is a praised method of teaching [9,35-37]. Studies by Soled et al [9] from the United States and Roskvist et al [37] from Australia highlight the advancement of virtual medical teaching formats away from the standard virtual web-based platforms. Soled et al discussed the use of interactive virtual medical committees consisting of 500 medical students with the aim to educate or partake in community activism to help with and understand the global pandemic, whereas Roskvist et al documented the replacement of normal clinical attachments with interactive e-learning placements in Australia. The results of the study by Roskvist et al [37] showed web-based learning to be as effective as traditional teaching. Virtual teaching within the United Kingdom mirrored virtual learning in Australia and America; more interactive and advanced technological forms of virtual learning were found to increase student engagement and focus [35]. Italy reported that virtual learning is an effective way to teach medical students during a time of crisis [36].

In stark contrast, developing countries such as Nepal, Iran, and Brazil negatively reported on the effectiveness of virtual teaching due to poorly funded and inadequate infrastructures for virtual learning [18,24,29,30]. Studies conducted in Nepal demonstrated apparent barriers to virtual teaching, including lack of knowledge on how to operate virtual platforms by staff and students alike, difficulties finding a quiet environment to study, technical challenges, reduced student engagement due to the use of self-directed learning with limited interactivity, and mental distress from social isolation [18,30]. In Iran, due to a lack of preparation of virtual learning resources, not all age groups within the medical curriculum had access to virtual teaching materials; also, medical education facilities experienced difficulties virtualizing different aspects of the medical course [28]. Finally, Carvalho et al [29] published a letter from Brazil documenting the challenges faced by the medical education system in Brazil. Similarly, the reported challenges to virtual teaching primarily arose due to a lack of investment. Moreover, Carvalho et al [29] documented the important points that not all students within Brazil have access to digital technology and some may be socially vulnerable, which further inhibits learning away from the university.

### Mental Well-Being of Students During the COVID-19 Pandemic

Virtual teaching for medical students is novel, and the distinct lack of social interaction may increase feelings of isolation, anxiety, and boredom [16]. The lack of physical, mental and social support from peers and institutions during this time may prevent learning [22] as a result of decreased motivation, lack of social engagement, decreased personal assessment of quality of life, and increased stress levels [16.] Feelings of isolation may further contribute to social withdrawal and cause a lack of student participation with virtual teaching resources [30]; in turn, the factors mentioned above may decrease academic performance [16,30]. A study conducted in Italy recognized that while short-term virtual learning is effective, long-term virtual learning would have significant negative effects on students, tutors, and administrative staff [36].

A study by Hodgson et al [42] discussed the use of virtual pastoral support to help students through this unsettling time. Virtual pastoral support was conducted through smartphones or computers at times that were convenient to the students; student engagement with this service was in high demand and student feedback was positive, noting that this support offered relief from stress and respite from studies. Moreover, the study highlighted that staff offering pastoral support may be less likely to interact with students virtually due to universities previously discouraging social media interaction and mobile phone contact with students [42].

### Limitations of the Review

Limitations to be considered in this review include its preliminary and exploratory direction; the literature available for review was restricted due to the emerging nature of COVID-19. In turn, this limitation governed a wider set of inclusion criteria and allowed the acceptance of all types of manuscripts within the review, which may reduce the acceptability of the results to a broader population. Moreover, PubMed was the only legitimate scientific database used, with Google Scholar providing supplemental searches. This limitation raises concerns that some papers relevant to the topic may have been missed. In addition, in the literature analyzed, many studies had small sample sizes; this may decrease the reliability of the findings. A further limitation of this review was the lack of studies that incorporated student perception of traditional teaching in comparison to virtual teaching.

### Conclusions and Recommendations

Virtual teaching for medical students has enabled medical education to continue despite the effects of the pandemic. The COVID-19 outbreak has provided medical education faculties with the perfect opportunity to develop and further the application and effectiveness of virtual learning for medical students. Medical education faculties should embrace the transition to virtual teaching and continue to develop web-based materials, such as secure web-based assessments and resources with increased student interactivity, to ensure that the most effective and suitable teaching is delivered. Virtual teaching requires significant investment from institutions, and many education faculties worldwide are struggling; institutions should actively seek to share web-based learning materials to improve content and accelerate student learning. Technical challenges and security concerns are inevitable barriers to virtual learning; students and staff members alike should strive to minimize these barriers.

## Abbreviations

PRISMA: Preferred Reporting Items for Systematic Reviews and Meta-Analyses
teleOSCE: teleconferencing objective structured clinical examination
